# Diagnosis and Treatment of Local Allergic Rhinitis

**DOI:** 10.3390/pathogens11010080

**Published:** 2022-01-09

**Authors:** Tetsuya Terada, Ryo Kawata

**Affiliations:** Department of Otolaryngology, Osaka Medical and Pharmaceutical University, 2-7 Daigakumachi, Takatsuki 569-8686, Japan; ryo.kawata@ompu.ac.jp

**Keywords:** allergic rhinitis, nasal allergen provocation tests, diagnosis, local allergic rhinitis

## Abstract

Some patients with chronic rhinitis have a positive nasal allergen provocation test (NAPT) without systemic IgE sensitization by skin prick tests or serum allergen-specific IgE (sIgE). This novel concept is called local allergic rhinitis (LAR) and affects children and adults worldwide, but is underdiagnosed. LAR is not just the initial state of allergic rhinitis (AR), it is a unique form of chronic rhinitis that is neither classical AR nor non-AR. Many of the features of AR and LAR are similar, such as a positive NAPT, positive type 2 inflammatory markers, including the nasal discharge of sIgE, and a high incidence of asthma. A differential diagnosis of LAR needs to be considered in patients with symptoms suggestive of AR in the absence of systemic atopy, regardless of age. The diagnostic method for LAR relies on positive responses to single or multiple allergens in NAPT, the sensitivity, specificity, and reproducibility of which are high. The basophil activation test and measurement of IgE in nasal secretions also contribute to the diagnosis of LAR. Treatment for LAR is similar to that for AR and is supported by the efficacy and safety of allergen exposure avoidance, drug therapy, and allergen immunotherapy. This review discusses current knowledge on LAR.

## 1. Introduction

Chronic rhinitis may be divided into two groups: allergic rhinitis (AR) and non-allergic non-infectious rhinitis, often simplified as non-AR (NAR) [1,2]. Due to its increasing incidence worldwide as well as its impact on quality of life, school performance, and productivity at work, AR has become an important public health issue [3]. Patients with allergies are identified by skin prick testing or the presence of allergen-specific IgE in serum [4,5]. Patients with AR test positive for at least one of these two diagnostic assessments of atopy [1], whereas non-AR individuals test negative for both [2]. The simple classification of chronic rhinitis into AR and NAR appears to be limited because it does not consider the form of rhinitis in which allergen-specific IgE produced locally in the nasal mucosa contributes to pathogenesis. The term local LAR has been proposed to describe Th2-type nasal mucosal inflammatory diseases in which antigen-specific IgE antibodies are produced locally in the nasal mucosa, the nasal allergen provocation test (NAPT) is positive, and systemic atopy is not proven [6]. We herein discuss the clinical implications of local allergy with a focus on the management of NAPT-positive patients without atopic rhinitis.

## 2. Epidemiology

LAR develops in a specific number of patients with chronic rhinitis, irrespective of nationality, ethnicity, or age [7,8,9]. Two recent systematic reviews and meta-analyses [10,11] showed the data from 3400 patients and healthy controls reporting a 24.7% probability of a positive NAPT in rhinitis patients that were negative for both skin prick test and serum sIgE. In a study on 648 patients with non-atopic rhinitis, nasally secreted IgE (sIgE) was detected in 10.2% of all patients and in 19.8% of those with a history of allergies [11].

The prevalence of LAR was previously suggested to be higher in Mediterranean countries (Portugal, Spain, Italy, and Greece) than in Nordic countries [12]. Furthermore, the prevalence of LAR due to house dust mites (HDM) was lower (<20%) in Asian countries than in Western countries, suggesting a higher prevalence of LAR (range 36.7–66.6%) in the latter than in the former [13,14,15,16].

## 3. Definition of Disease Concept and Etiological Classification

Nasal mucosal findings, nasal symptoms, the skin prick test, and the presence of antigen-specific IgE antibodies in serum have been used to classify non-infectious rhinitis as AR or NAR. However, after the establishment of a definition for LAR, these systemic tests were clearly limited because of their inability to accurately detect allergic inflammation in the nasal cavity. Therefore, a new etiological classification has been proposed for rhinitis. The term LAR was suggested by Rondón et al. [6] as a disease concept with Th2 inflammation of the nasal mucosa and the local production of antigen-specific IgE antibodies, but without any evidence of systemic atopy.

LAR has been attracting increasing interest in the last 15 years, and it is a term that is applied to patients with negative allergy skin and blood tests, but with a history suggestive of allergic sensitization and local evidence of atopy diagnosed by sIgE in nasal secretions, a positive nasal allergen challenge, or both, and who respond well to allergen-specific immunotherapy [17,18].

Concerning the endotype, LAR is a type 2 inflammatory disease that is caused by a localized allergic reaction in the nasal mucosa [19,20,21].

## 4. Pathophysiology of Local AR

The basic pathogenesis of LAR involves the localized production of antigen-specific IgE antibodies in the nasal mucosa and the completion of the antigen-antibody reaction locally. Previous studies revealed the localized production of sIgE in the nasal mucosa of patients with AR [22,23,24,25]. Furthermore, the nasal secretions of between 20 to 40% of NAPT-positive patients without systemic sensitization contained sIgE [20,21,26,27,28]. B cells in the nasal mucosa have been shown to express epsilon germ-line gene transcripts and mRNA for the epsilon heavy chain of IgE [29]. In situ hybridization revealed a type 2 inflammatory pattern, with an increased number of IgE + B cells, mast cells, and eosinophils, in patients with negative skin tests [19]. Although the mechanisms underlying the disease concept of LAR and AR have not yet been elucidated, a Th−2 lymphocyte and IgE antibody-mediated inflammatory reaction in the nasal mucosa of patients with LAR has been demonstrated. The mast cells and eosinophils of patients with LAR were found to be immediately activated in the nasal mucosa, releasing the characteristic inflammatory mediators tryptase and eosinophil cationic protein (ECP) [6].

Antigen-specific IgE antibodies have been observed in the nasal mucosa 24 h after NAPT, and are regarded as the basis for the localized production of antibodies in the nasal mucosa [28,30].

## 5. Clinical Phenotypes of LAR

Some of the clinical features of LAR and AR are similar.

Patients with LAR are generally young, non-smoking women with persistent, perennial symptoms of moderate to severe rhinitis that are often associated with complications including conjunctivitis and asthma [9].

The most frequent symptom is an itchy and watery nasal discharge commonly triggered by HDM [9]. The prevalence of LAR is higher in young adults [31]; however, children [9,13,30,32] and the elderly [33] are also affected. Furthermore, patients with LAR were found to be significantly younger than those with AR, and exhibited more severe symptoms as well as a family history of atopy [10,31].

## 6. Diagnosis

**Figure 1:**
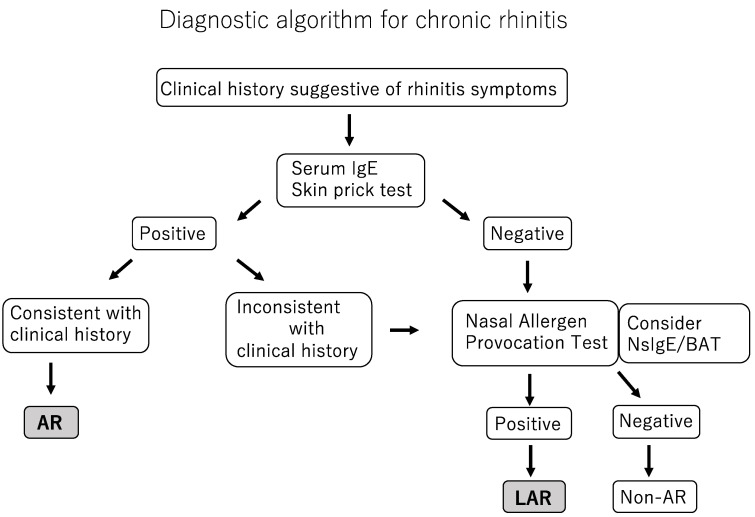
Diagnostic algorithm for chronic rhinitis.

Diagnostic algorithm for chronic rhinitis is shown in Figure 1.

- NsIgE: Nasal secretion IgE;
- BAT: Basophil activation test;
- AR: Allergic rhinitis;
- LAR: Local Allergic rhinitis;
- Non-AR: Non-allergic rhinitis.

LAR is diagnosed based on a detailed history, medical interview, a nasal allergic reaction by NAPT, and the exclusion of chronic sinusitis with or without nasal polyps in patients with a negative skin prick test and undetectable sIgE [34,35]. NAPT and the identification of antigen-specific IgE antibodies in nasal secretions are central to confirming a diagnosis of LAR.

Since the measurement of IgE antibodies in peripheral blood and antigen identification testing by skin prick tests are inadequate diagnostic tools for LAR, difficulties are associated with reaching an accurate diagnosis and, thus, the assessment of local responses in NAPT is required [27].

NAPT, the detection of sIgE in the nasal cavity, and the basophil activation test (BAT) are helpful diagnosis. NAPT is the current gold standard test for the diagnosis of LAR, but BAT and nasal sIgE test have the limitation that are difficult to use in clinical practice, and nasal sIgE test shows low sensitivity and inconsistent results [9,20,21,27,36,37]. Although NAPT may be used to differentiate between allergic (AR and LAR) and nonallergic disease, a saline test needs to be performed before NAPT to exclude non-specific hypersensitivity [6,9,36,38,39]. NAPT, a highly sensitive diagnostic method, may be conducted on children. Several standardized allergen solutions, either ready-to-use solutions or freeze-dried lyophilizates, are produced by different companies [40].

NAPT is considered to be positive when symptom severity markedly increases or combined objective and symptom measurements are moderately elevated [41].

The measurement of sIgE in nasal secretions is a non-invasive method for the diagnosis of LAR with high specificity, but low sensitivity (22–40% of responses) [20,21].

A previous study demonstrated the diagnostic accuracy of nasal sIgE for LAR in 212 children with chronic rhinitis; 14 had nasal sIgE > 0.35 kU/L and 12 were diagnosed with LAR based on significantly higher nasal sIgE than controls and a positive NAPT [42].

BAT has a sensitivity of 50% and specificity of >90% for *Dermatophagoides pteronyssinus* [43] and a sensitivity of 66% and specificity of >90% for *Olea europaea* [44], which is useful for reaching a definitive diagnosis of LAR.

## 7. NAPT Procedure

NAPT is one of the most important tests for the diagnosis of LAR.

Members of the EAACI Task Force reviewed the evidence based on systematic reviews involving NAPT over the past few years and proposed a method for standardizing the NAPT procedure in clinical practice [41]. The Task Force team proposed the use of a standardized test solution, with two puffs (0.1 mL per nostril) of bilateral spray, and subjective and objective assessment of the clinical outcomes. This technique aims to cover the mucosa of the inferior and middle portion of the nasal mucosa with the test allergen.

## 8. LAR and Asthma

Concomitant asthma symptoms were previously detected in 20–47% of patients with LAR [20,21]. Another study demonstrated that 50% of patients with LAR had a positive methacholine test and were diagnosed with asthma [45]. Among patients with AR and NAR, 83.3 and 57.9%, respectively, were diagnosed with asthma. Furthermore, the bronchial allergen challenge (BAC) was positive in 28.8 and 83.3% of patients with LAR and AR, respectively, but was negative in NAR patients and healthy controls [45].

After exposure to an allergen, a significant increase was noted in airway hypersensitivity testing with methacholine, and the numbers of eosinophils and monocytes in sputum were significantly elevated [45].

Significant increases were also detected in eosinophils, monocytes, and ECP in the sputum of BAC+ patients with and without atopy, but not in that of BAC-patients [45].

## 9. LAR Treatment

### 9.1. Pharmacological Treatment

The administration of oral H1-antihistamines to prevent new sensitization is not recommended for young children with nasal allergy and/or a family history of allergy mainly due to the risk of side effects and the lack of sufficient evidence to show reductions in the risk of developing new sensitization [46].

Similar to patients with AR, those with LAR respond well to topical nasal corticosteroids and oral antihistamines [20,21].

Oral antihistamines and intranasal corticosteroids are mainstay drugs for the treatment of AR [47]. Clinical experience suggests that these drugs are equally effective in patients with LAR and those with AR, and this may be attributed to their common clinical and pathophysiological features, such as eosinophilic rhinitis and reactivity to allergens.

It currently remains unclear whether oral antihistamines or nasal steroids are therapeutically effective for patients with LAR; however, a relationship between LAR and histamine metabolites was recently demonstrated in a cluster analysis of rhinitis endotypes [48].

### 9.2. Immunotherapy

Rondón et al. [17] examined the effects of subcutaneous immunotherapy (SCIT) on LAR by dividing patients with LAR sensitized to grass pollen into two groups: a group receiving preseasonal grass-specific SCIT for 6 months and rescue medication in spring, and a control group receiving only rescue medication. The findings obtained showed that SCIT reduced symptoms in patients with LAR.

For the primary outcome, the SCIT group showed a significant improvement in nasal tolerance compared with the control group (*p* = 0.001), with significantly higher threshold concentrations of grass pollen in NAPTs after 6 (*p* = 0.001) and 12 (*p* = 0.001) months of treatment, and 3 patients had negative NAPT responses.

Secondary outcomes were symptom and medication scores, medication-free days, and severity of LAR symptoms. In the active group patients reported a clinical improvement in the following spring, with a median reduction in average daily rhinoconjunctivitis symptom and rescue medication scores of 45% (*p* = 0.001) compared with the control subjects.

A 2-year randomized, double-blind, placebo-controlled clinical trial (RDBPCT) of SCIT for *D. pteronyssinus* (DP-SCIT) [18], a 2-year RDBPCT (Phl-SCIT) on *Phleum pratense* [49], and a 2-year RDBPCT (Bet-SCIT) that tested pollen from *Betula verrucosa* [50] provided supportive evidence for these findings. Furthermore, these studies demonstrated that SCIT exerted both short-term and sustained clinical effects for LAR [17,18,49,51].

SCIT also increased serum sIgG4 levels in patients with LAR in a volume-dependent manner, and this increase was attributed to IL-10-producing Treg and IgG4-producing Breg [52,53]; however, further studies are needed to assess the immunological effects of SCIT in LAR in more detail.

Collectively, these findings provide supportive evidence for the clinical efficacy of SCIT for LAR based on significant increases in tolerance to allergens and its positive effects on the quality of life of patients (Table 1).

### 9.3. Prevention Sensitization to New Allergens

Important treatment aims for AR with identifiable allergenic triggers include preventing the progression to asthma or other respiratory diseases and improving the quality of life of patients.

The concept of the allergic march has been proposed to describe the development of pediatric allergic diseases, and refers to allergic diseases progressing with multiple patterns of development from atopic dermatitis in infancy to bronchial asthma and AR. Infant-onset atopic dermatitis generally has a favorable prognosis, with remission or complete recovery being achieved by >90% of patients within several years. However, bronchial asthma occurs in 30–40% of these patients in infancy, and the inhalation of antigens (mainly mite antigens) contributes to the development of perennial AR by school age.

In a parallel group open study on AR and/or asthma patients with monosensitization to HDM, including 85 treated with allergen immunotherapy (AIT) and 62 with medication only, Inai et al. [54] showed the potential of AIT to prevent new sensitization, which suggested the importance of initiating AIT at an earlier age, particularly to prevent polysensitization in patients with rhinitis and monosensitization to HDM. The number of patients who did not show new sensitization after 5 years was significantly higher in the AIT group (75.3%) than in the control group (46.7%) (*p* = 0.002). Furthermore, sensitization to at least one new allergen was noted in 15 out of 21 patients in the AIT group, and to two or more new allergens in only 1 out of 6. In contrast, in the control group, sensitization to one new allergen was observed in 22 out of 33 patients, and to two or more new allergens in 11.

Although the mechanisms by which AIT reduced the risk of new sensitization in children have not yet been elucidated, AIT is known to alter the balance between TH_1_ and TH_2_ cells [55]. It also suppressed the production of interleukin (IL)-4 and IL-5 [56,57], promoted the production of interferon-γ [58], and decreased the number of inflammatory cells in the nose [59]. The induction of peripheral T cell tolerance by AIT is crucial for its efficacy and is attributed to increases in the levels of IL-10 and transforming growth factor-β produced by antigen-specific regulatory T cells. Important immune changes caused by AIT include increased tolerance to allergens and the development of specific tolerance by peripheral T cells in response to IL-10, which have been suggested to modify or prolong the natural progression of respiratory allergic diseases [60].

Although the underlying mechanisms currently remain unclear, the most effective early intervention for AR and LAR appears to be AIT, the initiation of which at earlier ages is recommended for the prevention of new sensitization.

## 10. LAR in Children

The prevalence of LAR in children ranges between 3.7 to 66.7%, and is lower in Asian countries (3.7–25%) [16,61,62] than in European countries (44.4–66.7%) [13,14,51]. HDM is the most common allergen in children with LAR worldwide. Ha et al. [62] performed NAPT with *D. pteronyssinus* on 145 children and diagnosed 5 with LAR. The largest study investigating LAR in children was performed by Krajewska-Wojtys et al. [51]; NAPT with *P. pratense, Artemisia vulgaris*, and birch pollens was performed on 121 patients aged between 12 and 18 years with confirmed NAR, but with typical seasonal nasal symptoms, and LAR was confirmed in 73 (52.5%) patients against *P. pratense, A. vulgaris*, and birch pollens in 17 (16.6%), 6 (5.9%), and 9 (8.9%) of patients, respectively.

The prevalence of LAR is high in children and slightly increases with age. Patients with LAR initially develop symptoms in childhood. Previous studies highlighted the importance of not only LAR as the main differential diagnosis of AR in children, but also target organ assessments by NAPT.

In a systematic review, nasal allergen reactivity was detected in 16.1% of children younger than 16 years of age with NAR [10,14,15,16,51,63].

## 11. Therapeutic Options

In addition to the classical subcutaneous and sublingual administration routes, the administration of allergens via intralymphatic, intradermic, or epicutaneous routes is now being investigated for airway allergies [64].

We previously demonstrated that intralymphatic immunotherapy was safe and effective for AR due to Japanese cedar pollinosis and also that clinical effects persisted for 1–2 years [65].

These routes have yet to be examined in detail in patients with LAR. A previous study reported that intranasal AIT was effective in an allergic asthma mouse model [59]. LAR is characterized by a local immune response in the nasal mucosa; therefore, the development of intranasal AIT strategies and comparisons of their clinical and immunological effects with those of SCIT are needed [17,18,49].

There is a lack of evidence for the efficacy of surgical treatment of LAR. As LAR is thought to be a local inflammation of the nasal mucosa, surgical treatment to reduce local inflammation in the submucosa might be effective against LAR. The reduction of the mucosal surface tissue reduces the point of contact with the allergen [66].

Surgical treatment might be another treatment option for LAR as scar tissue develops in the submucosa, destroying blood vessels and glandular structures and inhibiting regeneration through fibrosis.

## 12. Conclusions

A complete localized immune response in the nasal mucosa is the disease concept and definition of LAR. In the absence of systemic atopy, the differential diagnosis of LAR is currently based on the induction of allergic symptoms by antigen administration in the nasal mucosa and the presence of IgE antibodies in nasal secretions.

Difficulties are associated with confirming allergic inflammation locally in the nasal mucosa, starting from antibody production, sensitization, and the antigen-antibody reaction; however, this is the essence of the disease concept of LAR.

## Figures and Tables

**Table 1:** Studies on clinical efficacy of SCIT for LAR.

Author	Year	Country	Study Design	Study Group	Age (Year)	Allergen	Efficacy
Rondón, C. et al. [17]	2011	Spain	observational	20 LAR (seasonal)	adult	Phl	improve
Rondón, C. et al. [18]	2016	Spain	DBPCT	36 LAR (perennial)	adult	DP	improve
Rondón, C. et al. [49]	2018	Spain	DBPCT	56 LAR (seasonal)	18–55	Phl	improve
Bożek, A. et al. [50]	2018	Poland	DBPCT	28 LAR (seasonal)	18–76	Bet v1	improve

DBPCT, Double-blind placebo-controlled trial.
